# Trigeminal Nerve Control of Cerebral Blood Flow: A Brief Review

**DOI:** 10.3389/fnins.2021.649910

**Published:** 2021-04-13

**Authors:** Timothy G. White, Keren Powell, Kevin A. Shah, Henry H. Woo, Raj K. Narayan, Chunyan Li

**Affiliations:** ^1^Translational Brain Research Laboratory, The Feinstein Institutes for Medical Research, Manhasset, NY, United States; ^2^Department of Neurosurgery, Zucker School of Medicine at Hofstra/Northwell, Hempstead, NY, United States

**Keywords:** trigeminal nerve, trigeminal nerve stimulation, cerebral blood flow, cerebral perfusion, cerebrovascular resistance, neurogenic control of cerebral vasodilation

## Abstract

The trigeminal nerve, the fifth cranial nerve, is known to innervate much of the cerebral arterial vasculature and significantly contributes to the control of cerebrovascular tone in both healthy and diseased states. Previous studies have demonstrated that stimulation of the trigeminal nerve (TNS) increases cerebral blood flow (CBF) via antidromic, trigemino-parasympathetic, and other central pathways. Despite some previous reports on the role of the trigeminal nerve and its control of CBF, there are only a few studies that investigate the effects of TNS on disorders of cerebral perfusion (i.e., ischemic stroke, subarachnoid hemorrhage, and traumatic brain injury). In this mini review, we present the current knowledge regarding the mechanisms of trigeminal nerve control of CBF, the anatomic underpinnings for targeted treatment, and potential clinical applications of TNS, with a focus on the treatment of impaired cerebral perfusion.

## Introduction

Disordered cerebral perfusion, specifically inadequate perfusion leading to neurological injury, plays a major role in multiple disease processes including, but not limited to, acute ischemic stroke (AIS) (Prabhakaran et al., 2015), subarachnoid hemorrhage (SAH) (Ciurea et al., 2013; Baggott and Aagaard-Kienitz, 2014), and traumatic brain injury (TBI) (Vella et al., 2017; O’leary and Nichol, 2018). Cerebral blood flow (CBF) is normally determined by cerebral perfusion pressure (CPP) and cerebrovascular resistance (CVR). In turn, CVR is itself dependent on the degree of vasodilation, resistance, and blood viscosity (Fantini et al., 2016). The brain relies mostly on changes in vessel caliber and systemic arterial pressures to maintain CBF (Ter Laan, 2014). In pathologic states, these normal homeostatic mechanisms may be impaired, resulting in neuronal injury and death (Silverman and Petersen, 2020). As such, improving CBF, either by vasodilation or by elevating mean arterial pressure (MAP) to improve CPP, can be used to prevent ischemic injury and preserve at risk tissue. While numerous pharmacological strategies to improve cerebral perfusion have been put forth for the treatment of injured brains (Brott and Bogousslavsky, 2000; Prabhakaran et al., 2015; Lawton and Vates, 2017; Anghinah et al., 2018), most have failed to show even marginal benefit, emphasizing the importance of developing novel strategies.

Neurogenic control of CBF and autoregulation via the trigeminal nerve is of special interest as a mechanism that can be potentially harnessed to induce cerebral vasodilation, restore cerebral autoregulation, and improve cerebral perfusion. The trigeminal nerve is the largest cranial nerve, arising from the pons and dividing at the trigeminal ganglion into three branches (ophthalmic, maxillary, and mandibular) innervating most of the face, dura, and intracranial vessels (Kumada et al., 1977; DeGiorgio et al., 2011), with points easily accessible for percutaneous or transcutaneous manipulation. It also directly connects to vasomotor centers in the brainstem, chief among them the rostral ventral lateral medulla (RVLM) (Kumada et al., 1977; Goadsby et al., 1996; DeGiorgio et al., 2011). There have been promising initial reports on harnessing the trigeminal nerve via electrical stimulation (TNS) to restore homeostasis in the setting of disordered perfusion (Salar et al., 1992; Atalay et al., 2002; Shiflett et al., 2015; Chiluwal et al., 2017; Li et al., 2019). However, the study of the trigeminal nerve on cerebral vasculature has, up until this point, been mostly focused on its effect on disordered intra and extracranial CBF in the setting of migraine (Vecchio et al., 2018; Ashina et al., 2019; Iyengar et al., 2019). Furthermore, although TNS plays a pivotal role in the regulation of CBF, the effects of TNS on a perfusion-compromised brain remain unclear. In this review, we discuss the current understanding of mechanisms by which the trigeminal nerve controls CBF, the anatomic underpinnings of this control, and its potential in treatment of diseases of insufficient cerebral perfusion.

## Mechanisms Behind Trigeminal Nerve Control of Cerebral Blood Flow

**FIGURE 1:**
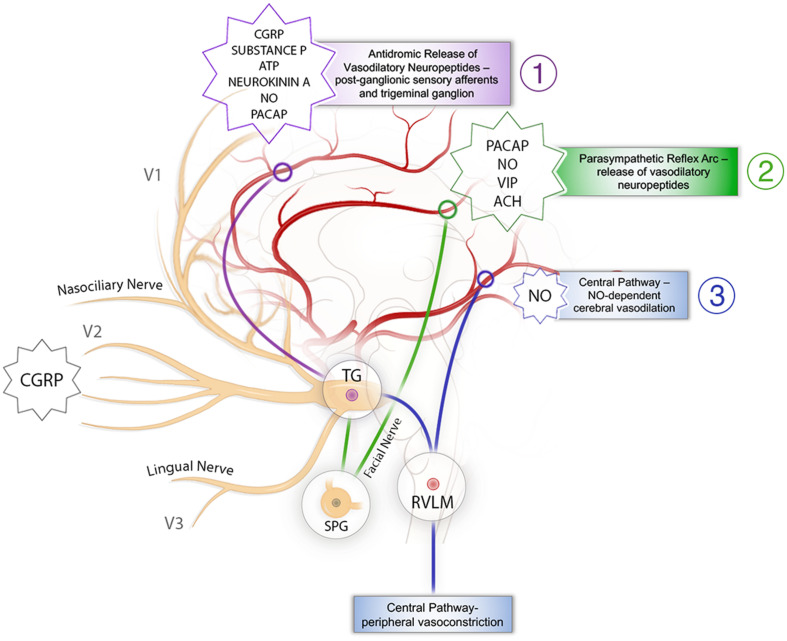
Schematic representation of the mechanisms behind trigeminal nerve control of cerebral blood flow. (1) The antidromic pathway: stimulation of sensory branches of the trigeminal nerve activates a pathway originating at the trigeminal ganglion that leads to antidromic release of neurotransmitters, vasodilation, and increases in CBF. It is presented in purple. (2) The trigeminal parasympathetic pathway: stimulation of sensory afferents from the trigeminal nerve results in parasympathetic vasodilation of the cerebral vasculature via interactions with the facial nerve and SPG. It is presented in green. (3) The central pathway: activation of RVLM causes cerebral vasodilation as well as inducing increased MAP leading to increased CBF. It is presented in blue. ATP, adenosine triphosphate; CBF, cerebral blood flow; CGRP, calcitonin gene-related peptide; MAP, mean arterial pressure; NO nitric oxide; PACAP, pituitary adenylate cyclase-activating peptide; SPG, sphenopalatine ganglion; TG, trigeminal ganglion; RVLM, rostral ventrolateral medulla; VIP, vasoactive intestinal peptide.

Stimulation of the trigeminal nerve has a threefold effect on cerebral vasculature, with each individual action leading to increased CBF (Figure 1). There is an antidromic impulse from the trigeminal nerve itself, which induces release of vasoactive peptides onto the cerebral vasculature, a parasympathetic reflex arc driven by sensory afferents of the trigeminal nerve, and a direct, central effect mediated through the RVLM. Each of these will be described in brief.

### Antidromic Pathway

The sensory branches of the trigeminal nerve spread across most of the face and directly innervate much of the cerebral vasculature (Shankland, 2000). Stimulation of these sensory nerves activates a pathway originating at the trigeminal ganglion that leads to antidromic release of neurotransmitters, vasodilation, and increases in CBF (Goadsby et al., 1988; Mense, 2010; Goto et al., 2017). This antidromic pathway was clearly delineated in 1974, when, after applying a parasympathetic blockade, TNS continued to induce elevations in CBF (Lang and Zimmer, 1974). Calcitonin gene-related peptide (CGRP), an extremely potent vasodilator, is likely the neurotransmitter that drives this vasodilatory effect (Edvinsson et al., 1987). Given the high concentration of CGRP in the trigeminal ganglion, it is likely produced in the ganglion and then transported to the free nerve endings surrounding the cerebral blood vessels, driving vasodilation, decreasing CVR, and increasing CBF (Messlinger, 2018). Other vasoactive peptides, including pituitary adenylate cyclase-activating peptide (PACAP), nitric oxide (NO), substance P, adenosine triphosphate (ATP), and neurokinin A, have been identified in either the free sensory nerve endings or the trigeminal ganglion (Uddman and Edvinsson, 1989; Gulbenkian et al., 2001), and are released as co-transmitters (Goto et al., 2017). However, it seems that CGRP has the most robust effect (Goadsby, 1993). The focal vasodilatory effect of CGRP release may be a promising mechanism to address diseases of cerebral vasospasm, as evidenced by previously observed increases in CGRP and decreased vasoconstriction in a rat model of SAH with TNS (Li et al., 2021). Though previous clinical studies in humans have shown that CGRP infusion following SAH can normalize cerebrovascular tone (Juul et al., 1994), further applications have been limited by the side effects inherent in the systemic application of a vasodilatory agent (European CGRP in SAH study, 1992; Kokkoris et al., 2012).

### Trigeminal Parasympathetic Pathway

Given the extensive spread of the trigeminal nerve, it is unsurprising that it would intersect and overlap with other cranial nerves (Tubbs et al., 2005; Shoja and Oyesiku, 2014), potentially allowing stimulation of the trigeminal nerve to cross-stimulate these intersecting nerves. One such nerve is the facial nerve, whose parasympathetic branches intersect with the trigeminal nerve at the sphenopalatine ganglion (SPG) and potentially the brainstem (Tubbs et al., 2005; Nturibi and Bordoni, 2020). While TNS-induced release of CGRP increases CBF, previous animal models have demonstrated that inhibition of CGRP does not completely abolish the response (Goadsby, 1993), partially due to the trigemino-parasympathetic reflex arc. Stimulation of sensory afferents from the trigeminal nerve results in parasympathetic vasodilation of the cerebral vasculature via interactions with the facial nerve and SPG (Lambert et al., 1984; Goadsby and Edvinsson, 1993; Branston, 1995). Though immunohistochemical studies have demonstrated that CGRP-containing sensory neurons are found in the SPG (Csati et al., 2012), parasympathetic nerve fibers have been found to release vasoactive molecules including acetylcholine (Ebersberger et al., 2006; Shelukhina et al., 2017), vasoactive intestinal peptide (Edvinsson et al., 1988; Goadsby and Shelley, 1990), PACAP (Uddman et al., 1993; Zagami et al., 2014), and NO (Goadsby et al., 1996), but not the aforementioned CGRP. This does ultimately result in increased CBF, decreased CVR, and cerebral vasodilation, but not via the same operative vasodilator (Goadsby et al., 1984). Though it has been seen that facial nerve stimulation and activation of the SPG may be protective in cerebrovascular disease (Borsody et al., 2013, 2014; Borsody and Sacristan, 2016; San-Juan et al., 2019), this has not been harnessed through the trigeminal nerve. It is thought that the decrease in CVR subsequently protects against impaired perfusion and worsening ischemia, with studies suggesting a large role for the parasympathetic system in pathologic states (Hamel, 2006).

### Central Pathway

The third pathway originates in the trigeminal ganglion itself through its brainstem projections (McCulloch et al., 1999; Golanov et al., 2000; Panneton and Gan, 2014). Once blood vessels leave the Virchow–Robin space, they lose their extrinsic innervation and are subsequently governed by intrinsic innervation projecting mostly from subcortical structures (Hamel, 2006). The trigeminal ganglion projects directly to the RVLM, an important medullary nucleus associated with systemic vasomotor control (Golanov et al., 2000; Panneton and Gan, 2014). With stimulation of the trigeminal nerve, a group of cells in the RVLM activate and cause peripheral vasoconstriction and subsequent MAP increase via adrenergic signaling pathways, ultimately directing blood toward ischemia prone organs such as the brain (McCulloch et al., 1999). Activation of RVLM also induces elevations in CBF independent of systemic blood pressure (BP), cerebral oxygen consumption, and metabolic demand, in an NO-dependent manner, via intrinsic projections (Golanov and Reis, 1994; Golanov et al., 2000). As such, stimulation of the RVLM induces elevations in CBF both from peripheral shunting by increasing MAP (Dutschmann and Herbert, 1998; McCulloch et al., 1999), therefore increasing CPP via vasodilation of the cerebral microvessels through intrinsic neuronal projections (Golanov and Reis, 1994; Golanov et al., 2000). This mechanism takes advantage of both maintaining, and in fact increasing, systemic BP, while simultaneously reducing CVR to preferentially shunt blood to the brain and increase CBF and may be especially useful in certain pathologic states such as SAH or for the prevention of secondary injury in the setting of TBI.

## Neuroanatomical Basis of Trigeminal Nerve Control of Cerebral Blood Flow

Stimulation of the trigeminal nerve may be topographically oriented, with specific branches leading to increased CBF in specific regions of the intracranial arterial tree (Figure 1). The majority of the intracranial blood vessels and dura are innervated by the ophthalmic division of the trigeminal nerve, the maxillary division innervates the skin over the midface, and the mandibular division innervates the jaw as well as the tongue (Brown, 1997). Research has shown that stimulation of each division has differential effects on the CBF (Table 1).

**TABLE 1:** Effects of electrical stimulation of the trigeminal nerve on cerebral blood flow.

Stimulation target	Stimulation method	Study group	Stimulation parameters					Result of stimulation on CBF		Adverse events	Study
		
			Frequency	Pulse amplitude	Pulse width	Waveform	Burst width	Observation	Location		
Opthalmic division	Minimally invasive EA, supraorbital nerve	Awake, resting humans	100 Hz		0.25 ms		1 min	↑ CBF	Prefrontal cortex	No adverse events occurred	Suzuki et al., 2020
	Invasive, right nasociliary nerve	Anesthetized rats	3, 10, 30, and 60 Hz	5 V	0.5 ms	Square Monophasic	90 s	↑ CBF w/↑ frequency	Ipsilateral parietal cortex	No adverse events reported	Suzuki et al., 1990
	Invasive, nasociliary nerve	Anesthetized, paralyzed cats	0.5, 1.5, 10, and 20/s	100 uA	0.25 ms	Square		↑ CBF	Posterior parietal cortex	No adverse events reported	Edvinsson et al., 1998
	Non-invasive, transcorneal, nasociliary nerve	Anesthetized rats with SAH	30 Hz	3 mA	1 ms	Biphasic	30 s	↑ CBF ↓ CVR w/and w/out SAH	Middle cerebral artery	No adverse events reported	Atalay et al., 2002
	Non-invasive, nasociliary nerve	Anesthetized rabbits	10 Hz	1, 2, 3, 4, and 5 V	0.5 ms	Square	90 s	↑ CBF as a function of voltage (1–5 V)	Premotor cortex	No adverse events reported	Gürelik et al., 2004
	Subcutaneous	Anesthetized rats with MCAO	25 Hz	60 μA	0.5 ms	Rectangular Cathodal	75 min	Not stated for electrical	Parietal cortex	No adverse events reported	Shiflett et al., 2015
	Invasive and subcutaneous Intermittent	Anesthetized rats with TBI	25 and 100 Hz	1–3 V	0.5 ms	Rectangular Biphasic	1 min	↑ CBF and ↓CVR w/and w/out TBI	Ipsilateral cortex	No adverse events reported	Chiluwal et al., 2017
Maxillary division	Percutaneous, infraorbital nerve	Anesthetized rats	2 Hz	1.2 mA	1 ms		210 s	↑ CBF	Barrel cortex	No adverse events reported	Weber et al., 2003
	Percutaneous, infraorbital nerve	Anesthetized rats	0.25, 0.5, 1.0, 2.0, 3.0, 5, 8, 10, and 12 Hz	2 mA 1–6 mA (1 Hz)	0.1 ms	Square	60 s	↑ CBF as a function of frequency, up to 3 Hz	Barrel cortex	No adverse events reported	Just et al., 2010
	Percutaneous, infraorbital nerve	Anesthetized rats with HS	25 Hz	7 V	0.5 ms	Rectangular Biphasic	1 min	↑ CBF after hemorrhagic shock	ML: +2 mm; AP: −2 mm	No adverse events reported	Li et al., 2019
	Percutaneous, infraorbital nerve	Anesthetized rats	50 and 133 Hz	0.25–3 V	1 ms	Rectangular Biphasic	1 min	↑ CBF ↓ CVR ↑ CGRP	ML: +2 mm; AP: −1 mm	No adverse events reported	Li et al., 2021
Mandibular division	Invasive, cut end of lingual nerve	Anesthetized rats	1–30 Hz	1–30 V	2 ms		20 s	No effect	Parietal cortex	No adverse events reported	Ishii et al., 2014
	Invasive, cut end of lingual nerve	Anesthetized cats	10 Hz	5–40 V	2 ms		20 s	No effect on CBF	Frontal cerebral cortex		Sato et al., 1997
Ganglion	Invasive	Anesthetized cats	10/s	500 uA	0.25 ms	Square		↑ CBF	Frontal and parietal cortex	No adverse events reported	Goadsby and Duckworth, 1987
	Invasive	Anesthetized cats	0.5, 1, 2, 5, 10, and 20/s	250 uA	0.25 ms	Square	30 s	↓ CVR	Parietal cortex	No adverse events reported	Goadsby et al., 1997
	Invasive, distal end	Isolated canine brains attached to anesthetized canines	50 Hz	10 V	1 ms	Rectangular Square	20 ms	↑ CBF ↓ CVR	Global	No adverse events reported	Lang and Zimmer, 1974
	Invasive	Anesthetized pigs	45/s	2.5 V 75 A	0.2 ms		3 h	↑ CBF w/and w/out SAH	Global	No adverse events reported	Salar et al., 1992

### Ophthalmic Division (V1)

The ophthalmic division of the trigeminal nerve innervates most of the cerebral blood vessels and the dura, as well as the skin over the forehead. The nasociliary nerve, which originates from the ophthalmic branch of the trigeminal nerve, contains major vasodilatory innervation for the middle cerebral artery (MCA) (Suzuki et al., 1989; Hosaka et al., 2016), and its stimulation results in the release of vasoactive neuropeptides from the free nerve ending, such as PACAP, substance P, and CGRP (Atalay et al., 2002; Gürelik et al., 2004; Ayajiki et al., 2005). Clearly, stimulation of V1 induces CBF increases due to the activation of all three mechanisms discussed above (Goadsby, 1993). Interestingly, stimulation of the dura along the superior sagittal sinus leads to elevated CBF, which is relatively more than when the trigeminal ganglion alone is stimulated (Goadsby and Duckworth, 1987). Further, it has been demonstrated in the setting of experimental TBI that stimulation of the nasociliary branch of the ophthalmic division of the trigeminal nerve can increase both CBF and brain tissue oxygenation (Chiluwal et al., 2017), and TNS following SAH retains the effect of elevated CBF and decreased CVR (Atalay et al., 2002). Importantly, the findings of greater CBF and vasodilation have been preliminarily observed in humans, with demonstrations of increased vessel diameter with pain stimulation of the V1 territory (May et al., 2001) and increased CBF with electroacupuncture of the supraorbital nerve (Suzuki et al., 2020). Given that increases in CBF have been demonstrated in experimental pathological models, and an increase in CBF has been observed in healthy humans, it is not an unreasonable assumption that stimulation of V1 represents a promising therapeutic target.

### Maxillary Division (V2)

The maxillary division innervates the skin over the midface and upper lip and maintains projection to the medullary dorsal horn and RVLM (Panneton and Gan, 2020). Though few papers have addressed the role of the maxillary division of the trigeminal nerve in altering CBF (Li et al., 2019, 2021), previous research has demonstrated clinical utility of V2 stimulation in the setting of epilepsy (DeGiorgio et al., 2003, 2006, 2009; Pop et al., 2011; Gil-López et al., 2020). Li et al. (2019) demonstrated that stimulation of the infraorbital branch of the maxillary nerve leads to improved cerebral perfusion in the setting of central hypovolemia. In this animal model, stimulation of the infraorbital nerve induced MAP and CBF increases, leading to improved brain tissue oxygenation. Further, in later experiments (Li et al., 2021), the observed increase in CBF was shown to be mediated via vasodilation and associated with increases in brain CGRP levels. Through combined monitoring of intracranial pressure, MAP, and CBF, they also demonstrated that TNS is capable of maintaining targeted cerebral perfusion thresholds in addition to vasodilation. They further demonstrated in an animal model of SAH that constriction of the internal carotid artery (ICA), one of the cerebral vessels affected by SAH, was significantly improved with TNS treatment.

### Mandibular Division (V3)

The mandibular division provides sensation to the jaw, lower lip, and anterior two-thirds of tongue. It is the only division of the trigeminal nerve that has motor fibers which innervate the muscles of mastication (Shankland, 2001). In contrast to the above sections of the trigeminal nerve, whose stimulation results in increased CBF, stimulation of the mandibular division has an unclear effect on CBF, with electrical stimulation of the lingual nerve increasing blood flow within the ICA, common carotid artery, lower lip, and palate (Sato et al., 1997; Ishii et al., 2014; Lapi et al., 2014, 2016). While this increase in blood flow is mediated via vasodilation, and the mandibular division is known to cause the release of CGRP into the bloodstream (Hill and Elde, 1988; Tsai et al., 1988; Kudo et al., 2019), it is not clear whether the said vasodilation and CGRP release are present within the cerebral intracranial vasculature. That being said, physical mandibular extension (submaximal mouth opening) has been shown to cause NO-related pial arteriolar vasodilation driven by trigeminal nerve afferents, ultimately resulting in CBF increases (Lapi et al., 2014, 2016). In contrast, direct electrical stimulation of the lingual nerve has not been shown to increase CBF (Sato et al., 1997; Ishii et al., 2014). Though both methods stimulate the mandibular division, the differing stimulation methods and anatomical localization support the hypothesis that differential trigeminal stimulation results in geographically distinct vasodilatory effects.

### Trigeminal Ganglion

The trigeminal ganglion (Gasserian ganglion), located in the base of the skull, receives sensory input from all the branches of the trigeminal nerve and projects subsequently to numerous brainstem nuclei (Kumada et al., 1977; DeGiorgio et al., 2011). Direct stimulation of the trigeminal ganglion in experimental models has been found to lead to CBF increases and reductions in systemic BP (Lang and Zimmer, 1974; Goadsby and Duckworth, 1987; Salar et al., 1992; Goadsby et al., 1997). A frequency-dependent decrease in CVR and carotid flow has been observed when stimulating the ganglion, while stimulation of the superior sagittal sinus results in a resistance reduction within the cerebral circulation, with a negligible effect upon carotid flow (Goadsby et al., 1997). Given that stimulation of the ganglion led to CBF increases and BP reductions, the driver of elevated CBF was likely cerebral vasodilation rather than BP elevation. Stimulation of the trigeminal ganglion drives the release of CGRP from the free nerve endings of the trigeminal nerve (Iyengar et al., 2019), as well as direct release from the ganglion itself (Eftekhari et al., 2010). Even though all three branches of the trigeminal nerve feed into the same ganglion, they do not have the same effect upon stimulation.

## The Effect of Trigeminal Nerve Stimulation on Disordered Cerebral Perfusion

The trigeminal nerve plays a pivotal role in CBF regulation by inducing cerebral vasodilation as well as by increasing systemic BP. Given this unique ability, it is somewhat surprising that this mechanism has only recently been applied to disorders of the cerebral perfusion. While inhibition of CGRP has proven effective in the setting of migraine (Edvinsson et al., 2018), which is a disorder of aberrant vasodilation by trigeminovascular system, very few studies have applied this principle to impaired CBF. Pathologies such as AIS, SAH, and TBI, all demonstrate disordered cerebral perfusion (Ciurea et al., 2013; Baggott and Aagaard-Kienitz, 2014; Prabhakaran et al., 2015; Vella et al., 2017; O’leary and Nichol, 2018) and could potentially benefit from stimulation of the trigeminal nerve.

In the setting of SAH, there are only three articles which have addressed possible therapeutic application of TNS. One demonstrated that Gasserian ganglion stimulation following SAH increased CBF (Salar et al., 1992), another showed that nasociliary stimulation (V1) following SAH resulted in elevated CBF and decreased CVR (Atalay et al., 2002), and the last showed that infraorbital stimulation (V2) resulted in increased CBF and CGRP levels, decreased CVR, and dilated ICA (Li et al., 2021). Similarly, the current literature on TNS for the treatment of ischemic stroke is quite limited. In a rat model of AIS, the authors found that TNS induced elevations in CBF and decreases in CVR, ultimately resulting in decreases in ischemic lesion volume (Shiflett et al., 2015). Other studies have demonstrated that electro-acupuncture of the scalp, at points innervated by the trigeminal nerve, led to decreased infarct volume and improved neurological function in animal models (Wang et al., 2014; Zheng et al., 2020). Additionally, the one paper which assessed the effect of TNS immediately following TBI indicated that it led to increased CBF due to decreased CVR, resulting in increased brain oxygenation and decreased neuroinflammation (Chiluwal et al., 2017).

As opposed to the systemic administration of medication, electrical TNS may be ideal for the treatment of these various pathologies as it is generally safe, has very few potential side effects, with no reported significantly adverse events (Table 1), can be administered to the targeted area non-invasively, and can be interrupted at any time (Gildenberg, 2006; Krames et al., 2009; Famm et al., 2013; Riederer et al., 2015; Leonard et al., 2017; Redgrave et al., 2018; Chou et al., 2019). However, with electrical non-invasive stimulation, the stimulation parameters need to be optimized, and the ideal target region for stimulation must be determined (Table 1). Currently, pre-clinical studies exhibit variability in electrical stimulation parameters, with little to no consistency even within publications from the same group. Given that few studies to date have determined if TNS is purely an “on–off” phenomenon, or if voltage and frequency can be tailored to elicit specific responses, this research would be critically important in the clinical setting. Further, it is vital that research be completed into the determination of why and to what degree stimulation of different trigeminal branches results in different effects. Though it is quite possible that the functional separation of the branches is due to the wide physical territory it spans, and the variation in surrounding anatomical structures, it is also possible that the reason is far more complex and could complicate future applications. Therefore, it is clear that a great deal of further investigation is needed into the potential effects of trigeminal nerve stimulation, its role in modulating cerebral perfusion, the sensitivity of different stimulation targets, and whether these effects are indeed significantly cerebro-protective.

## Conclusion

Many studies to date have demonstrated the successful regulation of CBF by TNS (Figure 1 and Table 1), providing the basic framework upon which to build future investigations into its potential applicability to various disorders of cerebral perfusion. Stimulation of the trigeminal nerve clearly has a significant impact on cerebral perfusion in both normal conditions and pathologic states; however, the potential of this approach to improve the recovery of patients with disordered perfusion has yet to be explored. In order to harness TNS for clinical application in diseases of inadequate cerebral perfusion, it is important to establish a better understanding of the molecular, physiologic, and anatomic underpinnings of TNS, and to establish which parameters are optimal in each disease state.

## Author Contributions

TW and KP performed the literature search and compiled the manuscript. KS drew the figure and revised the manuscript. HW and RN critically reviewed and revised this review article. CL conceptualized the theme of the review and edited the manuscript. All authors contributed to the article and approved the submitted version.

## Conflict of Interest

The authors declare that the research was conducted in the absence of any commercial or financial relationships that could be construed as a potential conflict of interest.
